# Multiple gene polymorphisms analysis revealed a different profile of genetic polymorphisms of primary open-angle glaucoma in northern Chinese

**Published:** 2009-01-16

**Authors:** Li-Yun Jia, Pancy Oi-Sin Tam, Sylvia Wai-Yee Chiang, Ning Ding, Li Jia Chen, Gary Hin-Fai Yam, Chi-Pui Pang, Ning-Li Wang

**Affiliations:** 1 Beijing Tongren Hospital of the Capital Medical University, Beijing, China; 2 Department of Ophthalmology and Visual Sciences, Chinese University of Hong Kong, Hong Kong, China; 3 Peking University People's Hospital, Beijing, China

## Abstract

**Purpose:**

To evaluate the individual and interactive effects of polymorphisms in the myocilin (*MYOC*),**optineurin (*OPTN*), WD repeat domain 36 (*WDR36*), and apolipoprotein E (*APOE*) genes on primary open-angle glaucoma (POAG) in northern Chinese.

**Methods:**

Northern Chinese study subjects, 176 POAG patients and 200 controls, were recruited for screening of the coding exons and splicing regions of *MYOC*. Five single nucleotide polymorphisms (SNPs) in *OPTN* (M98K, R545Q, IVS5+38T>G, IVS8–53T>C, and IVS15+10G>A), one SNP in *WDR36* (IVS5+30C>T) as well as the *APOE* promoter and ε2/ε3/ε4 polymorphisms were also examined. Association analysis was performed by using χ^2^ analysis. High-order gene-gene interaction was also analyzed using the multifactor dimensionality reduction (MDR) method.

**Results:**

In *MYOC*, 22 variants were identified. Four of them were novel but found in controls only. The missense mutation, Val53Ala, is likely a glaucoma causing mutation, accounting for 0.6% of cases. No individual polymorphism in *OPTN*,* WDR36*, or *APOE* was associated with POAG. MDR analysis identified a best 6-factor model for POAG: *MYOC* IVS2+35A***>***G, *OPTN* Met98Lys, *OPTN* IVS5+38T***>***G, *OPTN* IVS8–53T***>***C, *WDR36* IVS5+30C***>***T, and *APOE* −491A***>***T.

**Conclusions:**

The association pattern between the genes, *MYOC*,* OPTN*, *WDR36*, and *APOE*, and POAG in northern Chinese is different from that of southern Chinese. Disease-causing mutations in *MYOC* accounted for a small proportion of northern Chinese POAG patients. Common polymorphisms in these genes were not associated with POAG individually but might interactively contribute to the disorder, supporting a polygenic etiology.

## Introduction

Glaucoma is a heterogeneous group of optic neuropathies characterized by progressive degeneration of the optic nerve that leads to irreversible loss of vision. It is the second leading cause of blindness worldwide, estimated to affect about 70 million people [1]. Primary open-angle glaucoma (POAG, OMIM 137760) is the major type of primary glaucoma in most populations. It is evidenced as a complex disorder with multiple risk factors, and genetic factors may play an important role in the etiology of this disorder.

So far, at least 22 genetic loci had been linked to POAG [2-4], and three genes have been identified for POAG from the reported loci, myocilin (*MYOC*, OMIM 601652) [5,6], optineurin (*OPTN*, OMIM 602432) [7,8], and WD repeat-domain 36 (*WDR36*, OMIM 609669) [9]. However, mutations in these three genes account for less than 10% of POAG cases. Therefore, it had been assumed that only a portion of POAG follows the classical Mendelian inheritance while others are caused by variants in several genes, each of which contributes minor effects to disease onset and pathogenesis [10-12]. Presumably, many different genes, each with allelic variations, contribute to the observed variability in a trait, with no particular gene having a single dominant effect [13]. It is likely that single nucleotide polymorphisms (SNPs) in a single gene contributes only a small effect to disease [14] while some genetic variations do not cause diseases individually but act through interaction with other genes. Recently, Park’s study [15] had revealed that *OPTN* could regulate the expression of *MYOC* primarily through the control of mRNA stability, indicating that interaction exists between the two glaucoma genes. In addition, apolipoprotein E (*APOE*; OMIM 107741) has also been reported to be a potent modifier gene and may interactively contribute to POAG [10,11]. These findings provided evidence for the multigenic characteristics of POAG.

To date, the reported findings on glaucoma genetics in Chinese are mostly based on southern Chinese of Hong Kong [2-4,11,16,17], while relevant genetic information from the northern Chinese was limited. Recently, however, a genomic analysis revealed that the genetic ancestries of Chinese can be divided into a southern and a northern group [18]. Therefore, in this present study, we evaluated the genetic association of *MYOC*, *OPTN*,* WDR36*, and *APOE* polymorphisms with POAG in a group of northern Chinese. The distribution patterns of the gene variants between the northern and southern Han populations were also compared to discern whether any differential distributions of gene variants exist.

Moreover, possible gene-gene interactions among the variants in these genes were also evaluated. Statistically, for gene-gene interaction analysis, logistic regression (LR) is a commonly used method in case-control studies. However, it had been suggested that LR is less powerful if individual variables did not have significant main effects. Only variables that contain an independent main effect will be included in the final model [19]. Moreover, LR is less suitable for a large number of polymorphisms as more analyzed parameters may lead to higher likelihood of false positive and false negative results [20]. In contrast, multifactor dimensionality reduction (MDR) is a nonparametric method, which is less problematic in dimensionality, and is useful for analyzing the interactions among a large number of polymorphisms [20-23]. Therefore, LR was used to analyze the pairwise gene-gene interaction if any two gene variants are found to have significant main effects on POAG. Otherwise, MDR was applied to explore the high-order gene-gene interaction that may be involved in the genetic architecture of POAG in the northern Chinese population.

## Methods

### Case and control study subjects

Unrelated POAG patients and control subjects were recruited from the Eye Center of Beijing Tongren Hospital (Beijing, China). All the subjects are Han Chinese. They came from Beijing or nearby areas, representing a northern Chinese group. Diagnosis of POAG was based on exclusion of congenital glaucoma and secondary causes (trauma, uveitis, or steroid-induced glaucoma), anterior chamber angle open (grade III or IV gonioscopy), optic disc changes (vertical cup-to-disc ratio greater than 0.5, disc hemorrhage, or thin or notched neuroretinal rim), and visual field changes according to Anderson’s criteria [24]. Visual acuity was determined by the Snellen eye chart, intraocular pressure (IOP) by applanation tonometry, and visual field by a perimeter (Humphrey Field Analyzer; Carl Zeiss Meditec, Dublin, CA) with the Glaucoma Hemifield test. Unrelated control subjects were recruited from people attending the Tongren Eye Center for conditions such as senile cataract, floaters, and itchy eyes. They were given the same ophthalmic examinations and were diagnosed not to have glaucoma or other major eye diseases.

In our study, we included subjects with juvenile onset open-angle glaucoma (JOAG) and adult onset POAG. Totally, there were 176 sporadic patients with POAG, 138 males and 38 females, with age at diagnosis ranging from 10 to 82 years (mean±SD: 38.92±16.33) and with the highest recorded IOP before treatment being higher than 21 mmHg. We included control subjects aged 60 or above as they are less likely to develop POAG later in their lives. There were 200 control subjects, 150 males and 50 females, with age ranging from 61 to 85 years (mean±SD: 69.41±5.97) and with the highest recorded IOP of less than 18 mmHg. The study protocol was approved by the Ethics Committee for Human Research of Tongren Hospital and Capital Medical University (Beijing, China) and adhered to the tenets of the Declaration of Helsinki. Informed consent was obtained from all study subjects after explanation of the nature and possible consequences of the study. Venous blood was obtained from the subjects and stored at −20 °C for less than two months before DNA extraction.

### Analysis of the *MYOC* sequences and single nucleotide polymorphisms in *OPTN*,* WDR36*, and *APOE*

Genomic DNA was extracted from 200 μl of whole blood using a QIAmp Blood Kit (Qiagen, Hilden, Germany). To detect any possible novel disease-causing mutations in *MYOC*, the three coding exons and adjacent sequences of *MYOC* were screened by polymerase chain reaction (PCR) followed by direct DNA sequencing with a BigDye Terminator DNA sequencing kit on a 3130XL analyzer (Applied Biosystems, Foster City, CA), using the same set of primers that were used in our previous study [17].

As for the other three genes, only polymorphisms that might be informative in POAG genetics were selected for this study. If any significant association was detected, further fine mapping or re-sequencing of that gene will be performed. Accordingly, five SNPs in *OPTN* (c.603T>A [M98K], c.1944G>A [R545Q], IVS5+38T>G, IVS8–53T>C, and IVS15+10G>A) were selected, as they had been found to have statistical association with POAG or have interaction with other variants of the disease. They were genotyped by direct sequencing according to the protocol of our previous studies [3,16]. In *WDR36*, IVS5+30C>T had been found to be significantly associated with POAG in Hong Kong Chinese (unpublished data), and therefore was investigated by direct DNA sequencing, using the same pair of primers (primer for the forward strand: 5′-TAG ATT AGT ATC TAA GTC TGT GG-3′ and primer for the reverse strand: 5′-TGT TAT TTA TAG ACA ACC CTC CA-3′). In *APOE*, the promoter polymorphisms (−491A>T, −427T>C, and −219T>G) and the ε2/ε3/ε4 polymorphisms in exon 4 (c.526C>T for ε2 and c.388T>C for ε4) were investigated by the TaqMan genotyping assays in an ABI Prism 7000 Sequence Detection System (Applied Biosystems) according to the manufacturer’s instructions. The accuracy of genotyping with TaqMan was evaluated by direct sequencing in about one quarter of randomly selected samples according to the protocol of our previous study [11]. Complete matching of results was obtained.

### Statistical analysis

Hardy–Weinberg equilibrium (HWE) was tested for each polymorphism by the χ^2^ test. Allele or genotype frequencies between patients and controls were compared by the χ^2^ test or Fisher’s exact test. Significant levels for multiple comparisons were corrected by the Bonferroni method. SPSS version15.0 software (SPSS Inc., Chicago, IL) was used.

Pairwise linkage disequilibrium (LD) estimation and expectation-maximization (EM)-based haplotype association analysis were performed for the variants in *MYOC*, *OPTN*, and *APOE*, respectively, using Haploview 4.0 [25].

LR analysis was used for gene-gene interactions only if the variants were detected to have significant main effects. The disease status was set as the dependent variable (POAG=1, control=0) and gene polymorphisms as the independent variables (homozygote=2, heterozygote=1, wild type=0). The LR model was built with parameters for the independent effects of both variants. If significant interaction was detected, stratified analysis will be used to verify the gene-gene interactions. The study subjects will be stratified according to the genotype of one gene followed by the analysis of another gene in a different stratum defined by the genotype of the former gene. Homogeneity of the odds ratios (ORs) in different strata will be tested by the Breslow-Day test.

High order gene-gene interaction models among all the polymorphisms in the four candidate genes were detected and characterized using the multifactor dimensionality reduction (MDR) method as proposed by Ritchie et al. [23]. A detailed explanation on the MDR method has been described elsewhere [23,26,27]. Among the set of best multifactor models, the combination of genetic factors that maximizes the testing accuracy and/or the highest cross-validation consistency (CVC) is selected and further evaluated using permutation testing. p values associated with each prediction error and cross-validation consistency were determined by the Sign Test (a nonparametric test implemented in the MDR software) and corrected by a permutation test. For the best model, the interaction dendrogram, which was generated by MDR, was used to confirm, visualize, and interpret the interaction model. The MDR analysis was performed by using the open-source MDR software package (version 1.0.0, freely available online at Computation Genetics Laboratory).

## Results

### Univariate analysis of individual polymorphisms in *MYOC*

The allele and genotype frequencies of the *MYOC* variants were summarized in Table 1. In total, 22 polymorphisms had been identified. All of them followed HWE. Association analysis showed that none of these polymorphisms was statistically associated with POAG (p>0.0023, Bonferroni corrected significance level). Four variants (Gln161Arg, Gly183Ser, IVS1+16G>T, and Asn428Ser) were novel. They were detected in controls only. In contrast, variants Gly12Arg, Val53Ala, and Thr353Ile were found only in POAG cases with Gly12Arg in three POAG patients and the other two each in one patient. All were heterozygous. According to Alward’s criteria [28], they were likely disease-causing mutations (DCMs).

**Table 1:** *MYOC* polymorphisms identified in this study.

**Location**	** ** **Sequence change**	**Codon Change**	**Allele frequency (%)**		**Genotype frequency**	
			**POAG (n=352)**	**Control (n=400)**	**POAG (n=176)**	**Control (n=200)**
Promoter	−126 T>C	-	0 (0)	1 (0.25)	0/0/176	0/1/199
Promoter	−83 G>A	-	16 (4.5)	30 (7.5)	2/12/162	3/24/173
Exon 1	c. 34 G>C	G12R*	3(0.85)	0 (0)	0/3/173	0/0/200
Exon 1	c. 57 G>T	Q19H	0 (0)	1 (0.25)	0/0/176	0/1//199
Exon 1	c. 136 C>T	R46X	2 (0.57)	3 (0.75)	0/2/174	0/3/197
Exon 1	c. 158 T>C	V53A*	1 (0.28)	0 (0)	0/1/175	0/0/200
Exon 1	c. 227 G>A	R76K	13 (3.69)	28 (7)	0/13/163	1/26/173
Exon 1	c. 369 C>T	T123T	2 (0.57)	1 (0.25)	0/2/174	0/1/199
Exon 1	c. 482 A>G	Q161R	0 (0)	1 (0.25)	0/0/176	0/1/199
Exon 1	c. 547 G>A	G183S	0 (0)	1 (0.25)	0/0/176	0/1/199
Intron 1	IVS1+16 G>T	-	0 (0)	1 (0.25)	0/0/176	0/1/199
Exon 2	c. 611 C>T	T204M	0 (0)	2 (0.5)	0/0/176	0/2/198
Exon 2	c. 624 C>G	D208E	1 (0.28)	3 (0.75)	0/1/175	0/3/197
Intron 2	IVS 2+35 A>G	-	69 (19.6)	84 (21)	9/51/116	7/70/123
Intron 2	IVS 2+172 C>A	-	4 (1.14)	1 (0.25)	0/4/172	0/1/199
Exon 3	c. 864 C>T	I288I	1 (0.28)	1 (0.25)	0/1/175	0/1/199
Exon 3	c. 927 G>A	Q309Q	0 (0)	1 (0.25)	0/0/176	0/1/199
Exon 3	c. 1041 T>C	Y347Y	0 (0)	1 (0.25)	0/0/176	0/1/199
Exon 3	c. 1058 C>T	T353I*	1 (0.28)	0 (0)	0/1/175	0/0/200
Exon 3	c. 1283 A>G	N428S	0 (0)	1 (0.25)	0/0/176	0/1/199
Exon 3	c. 1464 C>T	A488A	0 (0)	2 (0.5)	0/0/176	0/2/198
3′UTR	1515+73 G>C	-	3 (0.85)	1 (0.25)	0/3/173	0/1/199

*MYOC* polymorphisms identified by direct DNA sequencing in this study were shown it the table. The asterisk indicates that the change is a disease-causing mutation and was found in POAG patients. The ‘hyphen’ represents non-coding variation. The numbers under “Genotype frequency” are the counts of homozygotes, heterozygotes, and wild type.

### Distribution of single nucleotide polymorphisms in *OPTN* and *WDR36*

Genotypic frequencies of *OPTN* (Met98Lys, Arg545Gln, IVS5+38T>G, IVS8–53T>C, and IVS15+10G>A) and *WDR36* (IVS5+30C>T) followed HWE in cases and controls. No significant difference was detected between POAG cases and controls in the genotypic or allelic frequencies (Bonferroni corrected significance level, p>0.01; Table 2).

**Table 2:** *OPTN* and *WDR36* polymorphisms investigated in the present study.

** ** **Gene**	**Sequence change**	** ** **Codon change**	**Allele frequency (%)**		**Genotype frequency**	
			**POAG (n=352)**	**Control (n=400)**	**POAG (n=176)**	**Control (n=200)**
*OPTN*	c.603 T>A	M98K	39 (11.08)	48 (12)	3/33/140	3/42/155
*OPTN*	c.1944 G>A	R545Q	12 (3)	13 (3.25)	0/12/164	1/11/188
*OPTN*	IVS5+38 T>G	-	118 (33.5)	121 (30.25)	17/84/75	14/93/93
*OPTN*	IVS8 −53 T>C	-	27 (7.67)	24 (6)	1/25/150	1/22/177
*OPTN*	IVS15+10G>A	-	8 (2.27)	7 (1.75)	0/8/168	0/7/193
*WDR36*	IVS 5+30 C>T	-	134 (38.1)	170 (42.5)	22/90/64	33/104/63

Allelic and genotypic frequencies of the five candidate SNPs in the present study were shown in the table. As only one *WDR36* SNP was selected, this SNP was also presented in this table. The “-” represents non-coding polymorphism. The numbers under “Genotype frequency” are the counts of homozygotes, heterozygotes, and wildtype.

### Univariate analysis of individual polymorphisms in *APOE*

Genotypes of all the *APOE* polymorphisms, i.e., −491A***>***T, −427T***>***C, −219T***>***G, and the ε2/ε3/ε4 polymorphism, followed the HWE in both study groups. Their genotypic or allelic distributions were not statistically different between POAG patients and controls (p>0.0125, Bonferroni corrected significance level, Table 3).

**Table 3:** *APOE* polymorphisms investigated in the present study.

** ** **Polymorphism**	**Allele frequency (%)**			**Genotype frequency (%)**		
	** ** **Allele**	**POAG (n=176)**	**Controls (n=400)**	** ** **Genotype**	**POAG (n=176)**	**Controls (n=200)**
−491 A>T	T	8 (2.3)	13 (3.25)	TT	0 (0)	0 (0)
	A	344 (97.7)	387 (96.75)	TA	8 (4.5)	13 (6.5)
				AA	168 (95.5)	187 (93.5)
−427 T>C	C	25 (7.1)	36 (9.0)	CC	1 (0.6)	1 0.5 (0)
	T	327 (92.9)	364 (91.0)	CT	23 (13.1)	34 (17.0)
				TT	152 (86.3)	165 (82.5)
−219 T>G	G	93 (26.4)	117 (29.25)	GG	10 (5.7)	15 (7.5)
	T	259 (73.6)	283 (70.75)	GT	73 (41.5)	87 (43.5)
				TT	73 (52.8)	98 (49.0)
ε2/ε3/ε4	ε4	38 (10.8)	36 (9.0)	ε4/ε4	3 (1.7)	2 (1.0)
	ε2	34 (9.7)	35 (8.75)	ε2/ε4	5 (2.8)	4 (2.0)
	ε3	280 (79.5)	329 (82.25)	ε3/ε4	29 (16.5)	28 (14.0)
				ε2/ε3	25 (14.2)	29 (14.5)
				ε2/ε2	2 (1.1)	1 (0.5)
				ε3/ε3	112 (63.6)	136 (68.0)

Allelic and genotypic frequencies of the four *APOE* polymorphisms in case and control subjects were shown in the table. None of them was significantly associated with POAG.

### Haplotype association analysis for the variants in *MYOC*, *OPTN*, and *APOE*

For *MYOC*, LD analysis revealed one LD block, spanning from the promoter (−83G>A) to exon 1 (c.227G>A). Within this block, polymorphisms −83G>A and c.227G>A were the only two variants that had a minor allele frequency (MAF) greater than 1%. They were in strong LD (D’=0.974). The haplotype G-G, defined by the major alleles of these two SNPs, presented in 95.5% of cases and 92.2% of controls. It was not statistically associated with POAG. The minor haplotype A-A was not associated with glaucoma. IVS2+35A>G was the only other *MYOC* polymorphism that had a MAF greater than 1%. When it was included in haplotype analysis, none of the haplotypes, which were defined by the three SNPs, showed any significant association with POAG (data not shown).

For *OPTN*, only five common variants were genotyped, and no LD block was detected. We had tried every possible combination of these five SNPs to evaluate haplotype association but found no haplotype significantly associated with glaucoma (data not shown).

For *APOE*, only the three promoter SNPs and the ε2/ε3/ε4 polymorphism in exon 4 were genotyped, and no LD block was detected. Haplotype analysis showed that the common haplotypes (frequency>5%), which are defined by the three promoter SNPs (−491A***>***T, −427T***>***C, and −219T***>***G), were not associated with POAG. When the ε2/ε3/ε4 polymorphisms (c.526C>T and c.388T>C) were included in haplotype analysis, no common haplotype was found to be significantly associated with glaucoma.

### Gene–gene interaction analysis

**Figure 1:**
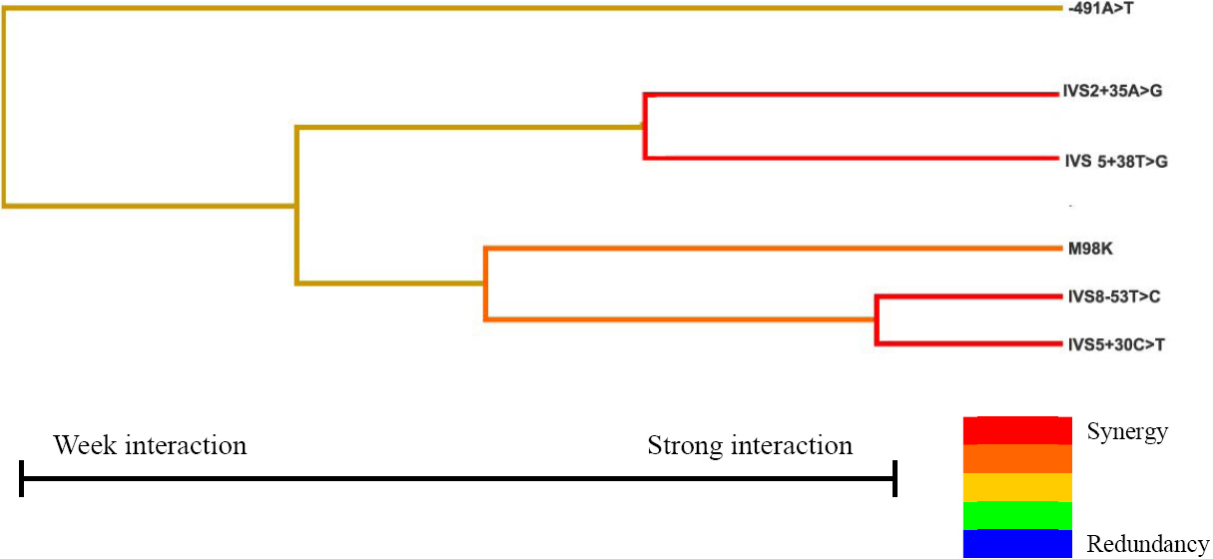
Interaction dendrogram for the six polymorphisms modeled by MDR. The colors comprise a spectrum representing a continuum from synergy to redundancy. The red color represents a high degree of synergy (positive information gain), orange a lesser degree, and gold represents the midway point between synergy and redundancy. On the redundancy end of the spectrum (not appeared in our model), the highest degree is represented by the blue color with a lesser degree represented by green.

Since we found no variant having strong significant association with POAG, we did not use logistic regression to build the interaction model. By using MDR analysis, six models were formed (Table 4), a best model that included six SNPs from all four genes was identified, including IVS2+35 A>G in *MYOC*; Met98Lys, IVS5+38T>G, and IVS8–53T>C in *OPTN*; IVS5+30C>T in *WDR36*; and −491A>T in *APOE*. The combination of these possibly interactive polymorphisms in the model gave a maximum CVC of 10/10 and a maximum testing accuracy of 0.5514. The p value associated with the prediction error and CVC was 0.0107 (p<0.001 after corrected by permutation test). Figure 1 illustrates the interaction dendrogram for this model. The hierarchical cluster analysis placed IVS5+30C>T (*WDR36*), IVS8–53T>C (*OPTN*), and Met98Lys (*OPTN*) on the same branch, and their closer position in the diagram clearly showed that the three SNPs may have a synergistic interaction effect on modulating risk of POAG. IVS5+38T>G (*OPTN*) and IVS2+35A>G (*MYOC*) were on another branch, revealing an interaction between them. −491A>T (*APOE*) was located on a different remote branch, suggesting that this SNP may have less of a relationship with other SNPs.

**Table 4:** Multi-locus interactions by the multifactor dimensionality reduction approach.

Best candidate model*	**Testing accuracy (%)**	**p value (sign test)**	**CVC**
−219T>G	43.61	0.999	5/10
IVS8–53T>C and IVS5+30C>T	52.5	0.377	7/10
IVS2+35A>G, IVS5+30C>T, and −219T>G	43.59	1.0	4/10
IVS2+35A>G, IVS5+30C>T, −219T>G, and 290C>T	47.53	0.945	9/10
A488A, IVS5+38, IVS5+30C>T, −427T>C, and −219T>G	49.16	0.623	10/10
IVS2+35A>G, M98K, IVS5+38T>G, IVS8–53T>C, IVS5+30C>T, and −491A>T	55.14	0.0107	10/10

Interaction models with one to six factors generated by the MDR program were shown in the table. The six-factor model with highest CVC (10/10) and maximum testing accuracy (55.14%) was selected as the best model in this study. The asterisk indicates that as the sample numbers in cases and controls are dissimilar in this study, the T value, which is the threshold ratio used to distinguish high-risk genotype combinations from low risk genotype combinations, was set to 0.88 (number of cases/number of controls, i.e., 176/200) while running the MDR program. CVC: cross-validation consistency.

## Discussion

For the first time, we have investigated the association between multiple genes and POAG in a northern Chinese population. In *MYOC*, we have detected four novel variants, Gln161Arg, Gly183Ser, IVS1+16G>T, and Asn428Ser, each in one control subject, but these variants were not found in POAG patients (Table 1). From published reports, *MYOC* mutations have been attributed to about 1%-4% of POAG cases, and this rate can be as high as 36% in JOAG families [29]. In the Chinese population of Hong Kong, which is southern Chinese, the prevalence of *MYOC* mutations is about 1.1%–1.8% in POAG cases [2]. In this present study, the non-synonymous polymorphisms, Gly12Arg, Val53Ala, and Thr353Ile, were found only in POAG cases but not in controls. According to Alward’s criteria [28], they were likely DCMs for POAG. However, a previous study on Hong Kong Chinese POAG patients showed that the Gly12Arg and Thr353Ile variants were not disease-causing; each of them was detected in four control subjects (0.7%)  [11]. Therefore, the remaining Val53Ala variant might be the only DCM for POAG in the northern Chinese group, accounting for only 0.6% of POAG cases. Such a mutation rate is lower than that reported in Caucasians and southern Chinese [2,29]. However, since the Gly12Arg variant was detected in 3 of the 176 POAG patients but none of the 200 healthy controls, we could not rule out its possibility of being a POAG-related variant in ethnic northern Chinese. A larger sample size is required to confirm its role on POAG. In addition, we found Gln19His in one control subject, which was previously suggested to be a DCM [11]. This subject was a 69-year-old female with normal fundus. She did not have a family history of glaucoma. Therefore, the Gln19His variant is unlikely a disease-causing mutation for POAG in northern Chinese. Apart from the rare variants, the three common polymorphisms (MAF>1%), −83G>A, R76K, and IVS2+35A>G, were not statistically associated with POAG, consistent with findings in southern Chinese in Hong Kong [11]. The allelic frequencies of these SNPs were also found to be similar between the northern Chinese of this study and the southern Chinese of Hong Kong (Table 5).

**Table 5:** Allele frequencies of *MYOC*, *OPTN*, and *APOE* variants in southern and northern Chinese.

**Location**	**Sequence change**	**Codon change**	**Allele frequency in HTG patients (%)**		**Comparison (p value)**	**Allele frequency in controls (%)**		**Comparison** ** (p value)**
			Southern* (n=588)	**Northern (n=352)**		**Southern (n=562)**	**Northern (n=400)**	
*MYOC*								
Promoter	−83G>A	-	37 (6.3)	16 (4.5)	0.26	50 (8.9)	30 (7.5)	0.44
Exon 1	c. 227 G>A	R76K	38 (6.5)	13 (3.7)	0.07	51 (9.1)	28 (7.0)	0.25
Intron 2	IVS2+35A>G	-	119 (20.2)	69 (19.6)	0.81	91 (16.2)	84 (21.0)	0.057
*OPTN*								
Exon 5	c.603 T>A	M98K	100 (17.0)	39 (11.1)	0.013	88 (15.7)	48 (12)	0.11
Exon 16	c.1944 G>A	R545Q	21 (3.6)	12 (3.0)	0.9	19 (3.4)	13 (3.25)	0.91
Intron 5	IVS5+38T>G	-	73 (12.4)	118 (33.5)	3.8×10^–15^	29 (5.2)	121 (30.25)	4.0×10^−26^
Intron 8	IVS8–53T>C	-	21 (3.6)	27 (7.7)	0.006	12 (2.1)	24 (6.0)	0.002
Intron 15	IVS15+10G>A	-	9 (1.5)	8 (2.3)	0.41	8 (1.4)	7 (1.75)	0.69
*APOE*								
Promoter	−491A>T	-	28 (4.8)	8 (2.3)	0.054	15 (2.7)	13 (3.25)	0.6
Promoter	−427 T>C	-	5 (0.9)	25 (7.1)	1.3×10^–7^	5 (0.9)	36 (9.0)	8.4×10^−10^
Promoter	−219 T>G	-	222 (37.8)	93 (26.4)	0.00037	187 (33.3)	117 (29.25)	0.19
Exon 4	ε2/ε4/ε3	R158C, C112R	63/39/486 (10.7/6.6/82.7)	34/38/280 (9.7/10.8/79.5)	0.076	48/52/462 (8.5/9.3/82.2)	35/36/329 (8.8/9.0/82.2)	0.99

Allelic frequencies of *MYOC*, *OPTN*, and *APOE* polymorphisms were compared between southern and northern Chinese. The asterisk indicates that the genetic data of the southern Chinese referred to our previous publication [11. In *MYOC*, only the three common SNPs (MAF>1%) were shown in the table, and only the frequencies of the minor allele of each polymorphism were shown in the table. The allelic frequencies of the polymorphisms were compared with the χ^2^ test. The p values were corrected by the Bonferroni method (p<0.05/13=0.0038 was considered statistically significant).

*OPTN* mutations had been reported to account for 1%–1.6% of sporadic Chinese POAG patients [11,16]. However, later studies reported no glaucoma causing mutations in *OPTN* among Caucasian and Japanese POAG patients [30-32]. In this study, the five SNPs that were selected to be genotyped had been reported to be either associated with POAG individually or act interactively with other genes on the disease [11,16]. After univariate analysis, none of them showed significant association with glaucoma. However, we found that the allelic distribution of the selected *OPTN* variants among this present study population was different from the southern Chinese population [11]. The allelic frequencies of the genotyped *OPTN* variants in the two populations are shown in Table 5. The exonic SNP, Met98Lys, presented a bit lower in the northern Chinese than that in the southern Chinese, but the difference was not statistically different (χ^2^=2.6, p>0.1 when comparing the allelic frequencies of this variant in the control group in the two studies, 12% versus 15.7% [11]). Another exonic variant, Arg545Gln, and the intronic variant, IVS15+10G>A, also showed comparable allelic distributions among the two Chinese populations. However, IVS5+38T>G, which was significantly associated with POAG in the southern Chinese, was found to be distributed drastically differently between the two populations. It occurred in 12.4% POAG and 5.2% controls in the southern Chinese. In contrast, in the northern Chinese, this SNP was detected in 33.5% of cases and 30.25% of controls, significantly higher than that in the southern Chinese after adjustment for multiple comparison (in cases, χ^2^=60.59, p=7.0×10^−15^; in controls, χ^2^=111.8, p=4.0×10^−26^). Another intronic SNP, IVS8–53T>C, was also found to have higher allelic frequencies in the northern Chinese, although the p values became borderline after Bonferroni correction (Table 5). Such difference in allelic distributions was also found for *APOE* −427T>C, which presented at significantly higher frequencies in the northern Chinese than the southern Chinese (Table 5). Such discrepancies can probably be explained by the presence of ancestry-related differences in allele frequencies between the northern and southern Chinese. However, only five SNPs in each of the two genes were investigated in the present study. Our data might have revealed only part of a discrepancy in the distribution pattern of *OPTN* and *APOE* polymorphisms. A more thorough investigation of the sequences of these genes is warranted to enable a more comprehensive comparison.

Recent studies suggested that *WDR36* defects may contribute to the glaucomatous disease process as a glaucoma modifier gene [33,34]. In a group of southern Chinese POAG patients, only one SNP in *WDR36*, IVS5+30C>T, showed significant association with POAG, and it is a common polymorphism (unpublished data). However, this SNP did not showed significant association with glaucoma in this study on the northern Chinese.

Findings of this present study suggest that the roles of *MYOC*,* OPTN*,* WDR36*, and *APOE* on the genetic architecture of POAG are different among northern and southern Chinese. To date, although variations in a variety of genes had shown statistical associations with glaucoma, these associations were often not replicable in other populations. Some of these SNPs may be of functional significance, and their frequencies may vary significantly between different ethnic groups. For example, three DCMs at *MYOC* (Gly252Arg, Gly367Arg, and Pro370Leu) were found in Asians and Caucasians, and three (Thr293Lys, Thr377Met, and Glu352Lys) were found in Africans and Caucasians. No single mutation was shared by all three ethnic populations, suggesting most DCMs exist in a specific ethnicity [29]. It may also suggest that a single gene locus may not cause glaucoma but act interactively with other gene variants [20]. Evidence for this hypothesis has been reported. Ishikawa et al. [35] found the *APOE* promoter polymorphism, −491A>T, interacted with *MYOC* −1000C>G (MYOC.mt1) to increase IOP in POAG patients, but the individual effect of MYOC.mt1 is unclear. Further, Funayama et al. [12] found that common polymorphisms in *OPTN* and olfactomedin 2 (*OLFM2*) may interactively contribute to the development of OAG in Japanese patients. In the Chinese population of Hong Kong, Fan et al. [11] detected three pairs of statistical interactions for normal tension glaucoma (between *MYOC* −83G>A and the *APOE* ε2/ε3/ε4, *MYOC* IVS2+35A>G and *APOE* −219T>G, as well as *OPTN* Arg545Gln and *APOE* ε2/ε3/ε4)) and two pairs of interactions for POAG (between *MYOC* Thr353Ile and *OPTN* IVS15+10G>A and between *OPTN* IVS5+38T>G and *APOE* −491A>T). In this present study, a best interaction model involving six variants in *MYOC*, *OPTN*, *WDR36*, and *APOE* had been identified for POAG in a northern Chinese population by using MDR. This finding suggested that these genes might act in concert to modulate the risk of POAG, although *MYOC* attributed a more dominant effect. According to the interaction dendrogram, the variants, *WDR36* IVS5+30C>T and *OPTN* IVS8–53T>C, are expected to have a strong synergistic interaction. *OPTN* Met98Lys has lesser synergistic interaction with both. Similarly, variants *OPTN* IVS5+38T>G and *MYOC* IVS2+35A>G also have strong synergistic interaction themselves but having lesser interaction with the three variants in the first branch. Moreover, *APOE* −491A>T may have the least degree of interaction with other variants. This dendrogram provided preliminary information about the interactive relationships among the variants in the model. However, one limitation of the MDR method is that it is difficult to evaluate the effects of each individual polymorphism in the models and to authenticate the combination of the risk alleles [36]. These issues still need to be addressed. Another limitation of this study is the lack of replication. As the interaction model detected in this study had not been identified in the southern Chinese or other populations, it should be evaluated in other Chinese populations.

In conclusion, we have for the first time investigated the roles of polymorphisms in *MYOC*, *OPTN*, *WDR36*, and *APOE* in POAG in a group of northern Chinese. The distributions of some variants were drastically different from that in the general southern Chinese population. The possibly disease-causing mutations in *MYOC* accounted for only a small proportion of northern Chinese POAG patients. The common polymorphisms in the candidate genes were not significantly associated with glaucoma, although a larger sample of northern Chinese should be required to unravel their contributions to POAG if their effects are mild. However, some of the common polymorphisms in these genes might interactively contribute to POAG, supporting a polygenic etiology of this common and complex disease.
